# Malnutrition and Fracture Healing: Are Specific Deficiencies in Amino Acids Important in Nonunion Development?

**DOI:** 10.3390/nu10111597

**Published:** 2018-10-31

**Authors:** Dennis M. Meesters, Karolina A.P. Wijnands, Peter R.G. Brink, Martijn Poeze

**Affiliations:** 1 Department of Surgery, Maastricht University Medical Center, P.O. Box 616, 6200 MD Maastricht, The Netherlands; n.wijnands@maastrichtuniversity.nl (K.A.P.W.); prgbrink@gmail.com (P.R.G.B.); m.poeze@mumc.nl (M.P.); 2 NUTRIM School for Nutrition and Translational Research in Metabolism, P.O. Box 616, 6200 MD Maastricht, The Netherlands

**Keywords:** fracture healing, nonunion, arginine, citrulline, nitric oxide, nitric oxide synthase

## Abstract

With the increasing incidence of fractures now, and in the future, the absolute number of bone-healing complications such as nonunion development will also increase. Next to fracture-dependent factors such as large bone loss volumes and inadequate stabilization, the nutritional state of these patients is a major influential factor for the fracture repair process. In this review, we will focus on the influence of protein/amino acid malnutrition and its influence on fracture healing. Mainly, the arginine-citrulline-nitric oxide metabolism is of importance since it can affect fracture healing via several precursors of collagen formation, and through nitric oxide synthases it has influences on the bio-molecular inflammatory responses and the local capillary growth and circulation.

## 1. Introduction

The absolute number of fractures will increase in the future due to rapid aging of the population and the associated incidence of osteoporosis [1]. Current estimations for a person in the general population on sustaining a fracture are 1 in 100 persons per year [2], with risks of suffering an osteoporotic fracture ranging between 13% and 50% [3,4]. The observed change in lifestyle in the older population will lead to people being more active until a higher age and increased sports-related fractures [5]. With increasing incidences in (different types of) fractures, the absolute number of complications during the fracture-healing process, such as nonunion development, will also show an increased prevalence [6]. Next to a drastically diminished quality of life for the patients, high socio-economic costs contribute to the problem of nonunion development [7]. Nutrition has a major influence on fracture healing, with observed fracture-healing impairment in the malnourished and undernourished population. In this review, we will focus on the influence of malnutrition on bio-molecular responses and subsequent complications during the fracture-healing process and nonunion development.

### 1.1. Normal Fracture Healing

To understand nonunion development, first normal fracture healing will be discussed. Fracture healing is a complex process of partially overlapping sequential phases, starting with an inflammatory response and hematoma formation caused by the tissue reactions on vascular and soft tissue damage [8,9]. The inflammatory phase is followed by the formation of a soft fibro-cartilaginous matrix consisting primarily out of fibroblasts and chondrocytes, providing primary mechanical stability at the fracture site [9]. The cartilaginous matrix acts as a template for the hard callus in the third stage of the healing process. During the osteogenic phase of primary bone formation, irregular woven bone is formed and vascularization towards the callus increases. Finally, after the primary bone formation, this irregular woven callus is replaced into lamellar bone, thus forming the original cortical and trabecular form of the bone [8,9,10].

### 1.2. Nonunion Development

In the healing process of five to ten percent of all fractures, difficulties occur resulting in or nonunion development of the fracture [11,12,13]. Although there is no definitive consensus [14], nonunions are clinically characterized by motion between the fracture parts and a persisting fracture line present on radiographic imaging. This finally results in the development of a synovial pseudo-arthrosis after 6 to 9 months after the initial trauma [15,16]. Traditionally, nonunions are classified either as hypertrophic or atrophic [16]. Hypertrophic nonunions usually are well vascularized and exhibit partial callus formation. However, due to the inadequate stabilization of the fracture parts, movement impedes the formation of a solid callus [16]. On the contrary, atrophic nonunions show low levels of callus formation and are usually considered to be of an avascular or metabolic origin hampering callus synthesis.

Risk factors for nonunion development are generally divided into fracture dependent or patient dependent [17]. Fracture-dependent factors include a lack of cortical apposition, the presence and degree of communition, displacement of the fracture [18], blood supply to the fracture region [19,20], presence of periosteal damage [21,22,23] and soft tissue damage associated with infection [24]. Logically, critical sized segmental defects also have a higher nonunion risk due to large segmental bone loss [25]. Patient dependent risk factors include age, gender, genetic disorders, metabolic diseases, smoking, non-steroidal anti-inflammatory drug (NSAID) use and the patient’s nutritional status [17,23,26] among others. As for age and sex, the distribution of nonunions has distinct peaks in males in the age category between 25 and 29 years. As for females, elderly aged around 75 years, where show a sharp incline starting after their 65th year [6]. This incidence reflects the epidemiology of fractures in men and women and their age distribution [27,28]. Metabolic diseases resulting in vitamin D deficiency, thyroid disorders and parathyroid hormone disorders are more often found in patients who development a nonunion. Medical treatment alone of these comorbidities can result in union of the fracture parts [29].

The nutritional status, especially malnutrition will be discussed in more detail in the next paragraphs in this review.

## 2. Proteins and Malnutrition in Fracture Healing

### 2.1. Influence of Collagens and Bone Morphogenetic Proteins on Fracture Healing

Proteins which are most abundant in bone and which have major influences during the fracture-healing process are the different types of collagen and the bone morphogenetic proteins (BMPs).

Collagen is the most abundant protein present in bones [30]. Until now, 28 types of collagen have been discovered, of which type I (Col I) represents 90% of the total collagen in the human body and which is the main component of the organic part of the bones. A normal type 1 collagen protein consists of so-called alpha-1 type I collagen chains and alpha-2 type 1 collagen chains which combined form a molecule of type I procollagen. Extracellularly, these molecules are processed and arranged into thin fibrils with the ability to cross-link with each other resulting in mature collagen fibers. These mature collagen fibers, three-dimensionally exhibit a triple helical-like structure, which prevents collagen from being broken down by enzymes, contributing to adhesiveness of cells and formation of the extracellular matrix [31]. Formation of abnormal and irregular collagen fibers can be the result of a vitamin C deficiency, since ascorbic acid is a cofactor for collagen synthesis. Presence of irregular fibers will result in a delayed healing of the fracture or possible formation of a decreased strength in the newly formed bone resulting in a higher chance of subsequent fractures [30,32]. In scaffolds which are used for tissue regeneration, collagen is often used since the in vivo stability but also the pore-like structure contributes to the adhesion of fibroblasts and osteoblasts [33].

Other types of collagen which are related to bone healing and bone formation are collagen X (Col X) and collagen XI (Col XI). Both Col X and Col XI are mainly found in (hypertrophic and mineralizing) chondrocytes and so play a role in formation of the soft cartilaginous fibroblastic matrix formed during endochondral ossification.

The second important player are the BMPs. During primary bone formation, BMPs, which are glycoproteins, are involved in mediating the process of osteoblasts synthesizing the mineralized callus [34]. BMPs first described by Marshall Urist in the 1960s [35] belong to the transforming growth factor (TGF) superfamily [36]. BMPs, are known not only to be active in growth and differentiation but also show high degrees of osteogenic potential in in vitro, as well as in animal and human in vivo research [37,38,39,40]. BMP signaling follows a time-dependent sequential cascade of chondrogenesis, osteogenesis, angiogenesis and the synthesis of extracellular matrix [41,42], allowing them to be used for influencing bone formation throughout the complete course of the fracture-healing process. Although a variety of approximately twenty BMPs have been identified and classified [36], until now, only recombinant human (rh) BMP2 and rhBMP7 (also known as osteogenic protein-1; OP-1) are used clinically in orthopedic and trauma surgery [43,44].

Numerous (genetic) studies have found a wide array of signaling pathways leading to different proteins which are involved in the fracture-healing process. One of the most intensively studied is the β-catenin-dependent Wnt signaling, which has been reviewed extensively before [45,46]. However, since this canonical Wnt signaling pathway mainly is involved in maintaining bone mass, it is mainly investigated within osteoporotic fractures as it might counter the bone volume inhibitory effects of overexpressed molecules as Dickkopf (Dkk) and Sclerostin (Scl) [47,48], this pathway is not further reviewed within this paper.

### 2.2. Nutritional Status and Fracture Healing

Older adults (>65 years) are at a higher risk of malnutrition [49]. Malnourishment is the physical condition in which a person’s food intake is either too low or high for one or more nutritional factors, or a misbalance between the nutritional factors is present. Associated with malnourishment is a lower body mass index (BMI) which also is correlated with an increased fall risk [50]. This increased fall risk may result in a higher risk for sustaining a fracture, hence it may contribute to the total number of nonunions developing in these trauma patients.

A poor nutritional state increases the risk for osteoporotic fractures [51] and also of nonunion development as osteoporosis leads to a reduction of osteoblasts and callus production [23,52]. Next to osteoporotic fractures, mal/undernourished patients tend to have more fall incidents when compared to non-malnourished patients [53]. The frailty of especially the elderly population undergoing surgery is associated with higher rates of mortality [54] and a longer hospital stay [55] and multiple readmissions [56,57]. In addition, the prolonged inactivity of patients after hospital admission and revalidation after a traumatic fracture can result in a substantial loss of skeletal muscle mass of up to 5% of total muscle mass within the first two weeks after trauma [58]. This sarcopenic state of the patients contributes to the anabolic process of bone formation during the healing period, resulting in a higher chance of nonunion development [59,60].

A vastly explored method for improving malnourishment is supplementation with different amino acids, which is investigated in animal testing [61,62,63,64] as well as in (hospitalized) patients [65,66,67,68], for a range of different conditions such as cancer, cardiac disease, sepsis, and liver metabolism, but also for its possible beneficial effects on post-surgical infection development [69] and in orthopedic diseases [70].

In geriatric trauma patients, the majority of essential and non-essential amino acids is known to be significantly decreased when compared to healthy geriatric control patients [71]. Already in 1976, lower ornithine concentrations were observed in patients with fractures after major trauma [72] when compared to control patients. These results are in line with the fact that ornithine is, through polyamine production, a precursor for collagen synthesis [73]. Underlining these findings is the fact that a deficiency of ornithine is contributing to the enhanced nonunion risk in multi-trauma, patients [17,23], which often also exhibit a malnourished state during the prolonged hospitalization and immobilization. Protein-depleted patients with a fracture of the hip showed higher prevalence of complications, a longer admission period in the hospital and a lower one year survival probability [74] when compared to non-depleted patients.

Next to essential amino acids [75,76], non-essential amino acids such as glutamine, arginine and their precursors possess beneficial anabolic properties which are essential during fracture healing [77].

Hughes et al. observed [78] an enhanced fracture and soft tissue healing, in rats were a closed femoral midshaft fracture was induced with subsequent intramedullary nailing and afterwards received anabolic dietary supplementation, consisting of proteins and the conditionally essential amino acids glutamine, arginine and taurine. Groups with high concentrations of proteins and the conditionally essential amino acids glutamine and arginine (among others) showed increased muscle mass and bone mineral density in the fracture callus after a healing period of six weeks when compared with animals that were fed a diet with low concentrations of proteins. In a comparable study [79], malnourished rats (protein-depleted) which underwent a closed femoral fracture, showed a callus primarily composed of fibrous tissue with decreased periosteal and endosteal callus size and decreased callus strength when compared with control animals which underwent a closed femoral fracture.

**Figure 1:**
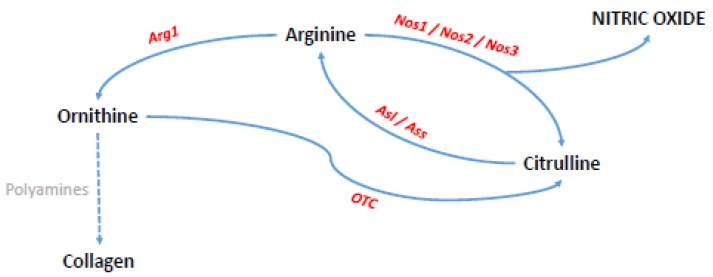
Schematic representation of the arginine-citrulline-nitric oxide metabolism. Arginine can be converted into citrulline by one of the isoforms of nitric oxide synthase (NOS1, NOS2 and NOS3). During the conversion, nitric oxide is produced. Citrulline can be converted back into arginine by the enzymes arginosuccinate synthetase (ASS) and arginosuccinate lyase (ASL). Conversion of arginine into ornithine is mainly via arginase 1 (Arg1). Ornithine acts as a precursor for collagen synthesis through conversion via several polyamine molecules. Along a second route, it can be converted into citrulline by the enzyme ornithine transcarbamylase (OTC).

The focus in most of these studies [75,76,77,78,79] is the semi-essential amino acid arginine. During normal physiological conditions, arginine is produced from conversion of citrulline by the two cytosolic enzymes arginosuccinate synthetase (ASS) and arginosuccinate lyase (ASL). The importance of arginine is due to it being the only precursor within the human which physiologically can be converted into nitric oxide. Citrulline exhibits a low dietary intake (13% of total arginine); however, 60–80% is contribute by the conversion of glutamine into citrulline in the enterocytes of the small intestine. A third way in which citrulline can be produced is degradation of ornithine via ornithine transcarbamylase. In Figure 1, a schematic overview of the arginine-citrulline-NO-metabolism is presented.

### 2.3. Influences of Nitric Oxide and Nitric Oxide Synthases on Fracture Healing

Several in vitro studies provide a solid scientific base for the possibilities of stimulating the arginine-nitric oxide (NO) metabolism with the interest of enhancing the fracture-healing process.

Stimulation of human osteoblasts derived from osteopenic patients with arginine has been shown to have a positive effect on proliferation, activation and differentiation and matrix synthesis [80], thus suggesting possibilities in prevention of osteoporotic fractures.

Previously, Chevalley et al. reported [81] on the influence of arginine on murine osteoblast-like cells. The supplementation of arginine increased the concentrations of IGF-1 (insulin-like growth factor-1) and the de novo collagen synthesis. As IGF-1 is a potent regulator of osteoblastic bone formation, supplementation of arginine might be a promising option in malnourished patients with osteoporotic fractures [82]. In combination with L-lysine supplementation of arginine, beneficial effects on proliferation were shown in osteoblast cultures from osteopenic rats [83], resulting in increased type 1 collagen formation.

In 2000, Diwan et al. reported [84] that nitric oxide (NO) had a role in fracture healing as it was expressed in callus tissue during fracture healing. Also, NO was known to play a critical role in other physiological processes such as wound-healing [85] and tendon healing [86]. Next to its role in fracture repair, NO is known to be involved in a wide range of musculoskeletal conditions such as inflammatory arthritis [87,88], osteoporosis [89], aseptic loosening of implanted prosthesis [90] and tendon healing [86,91].

After inducing a closed right femoral midshaft fracture in rats by three-point bending [92], the importance and presence of NO production in fracture healing was shown by the activity of calcium-dependent and -independent nitric oxide synthase (NOS1 and NOS3) activity that was detected in homogenized fracture callus by using a conversion assay of [^3^H]-arginine to [^3^H]-L-citrulline. In addition, immunohistochemical tests localized NOS2 presence in these rats at the junction of fibrous tissue and the cartilage front. On mRNA level, expression of NOS2 was present after 4, 7 and 15 days of fracture healing, whereas NOS1 and NOS3 were only expressed after 7 and 15 days.

Human fracture callus samples collected in patients undergoing open reduction and internal fixation of a fracture [84] NOS2 and NOS3 mRNA was present in 3- to 5-day old fractures. NOS1 was only present in a 90-day old fracture.

Rats fed the nonselective NOS inhibitor L-Nitroso-arginine methyl ester (L-NAME) via the drinking water *ad libitum* prior to surgery inducing an midshaft femoral fracture and during the follow-up period, showed an approximate 20% decrease in cross-sectional callus area and average mineral density when compared to rats fed the inactive enantiomer D-NAME [84]. During biomechanical testing, these NOS-inhibited rats showed a decrease in peak failure load, stiffness and energy required to break the healing femur. In addition, supplementation of an NO donor (NONOate derivative of carboxybutyl chitin) resulted in a 30% increase in cross-sectional area was shown when compared to L-NAME treated animals. However, rats which received L-NAME did not show a distinct change in trabecular bone formation rate [93].

These results are in line with studies investigating wound-healing responses, were addition of an NO donor increases skin wound-healing [94] whereas NOS2 gene deficient animals which showed decreased wound closure rates [95]. In fracture healing, deletion of the NOS2 gene in mice [96] showed a significant decrease in maximum energy absorption during biomechanical testing when compared to normal wild type mice. NOS2^−/−^ mice receiving NOS2cDNA directly at the fracture site by implantation of a gelatine sponge, showed normal energy absorption and an increased callus cross-sectional area.

In addition to this study, Zhu et al. reported on type specific and time-dependent expression of different NOS isoforms in the fracture-healing process [97]. In an open rat fracture model in which controlled femoral midshaft fracture was made and afterwards fixed with a 1.6 mm Kirschner wire, all NOS isoforms were expressed during the first 21 days of fracture healing. However, were NOS2 had its peak after 4–7 days, consistent with the inflammatory phase in the fracture-healing process [98]. NOS3 mRNA and protein were mainly expressed between 7–14 days, were osteoblastic differentiation and activity is at is maximum [99]. The neuronal NOS1 was found after 21 days during the remodeling phase of the fracture, indicating a lower importance in nonunion development since disturbances during the early and middle stages of fracture repair generally lead to nonunions.

Next to the distinctive temporal expression of the different NOS isoforms, an isoform dependent spatial localization is found in healing rat fractures [100]. The initially upregulated NOS2 is found mainly along the edge of the periosteal callus, close to the cortical bone and in areas of endochondral ossification. Endothelial NOS3 was primarily present in cells lining blood vessels and cells in the chondral region. Lastly, NOS1 showed a signal between the fibrous tissue and cartilage within the fibrochondral region of the healing fracture [97,100].

NOS2-derived NO production is important in bone formation by mediating the transduction of a mechanical stimulus into biological responses in bone [93,101,102]. When treating rats with the selective NOS inhibitor aminoguanidine, bone formation rate, mineral apposition rate and the percentage of mineralizing surface is significantly lower in the proximal tibial epiphysis when compared with control animals [93]. In addition, NOS expression is shown to correlate with new bone formation during distraction osteogenesis in rats [103].

In a tail-suspension model simulating hindlimb unloading in mice, the role of NOS2 in skeletal adaptation to acute increases was investigated [104]. Gene deficient NOS2 mice showed a decreased bone volume and bone formation rate after 7 days of tail suspension. During subsequent 14-day reloading, increases in bone formation and volume were abolished in NOS2 mice in comparison with control animals. Treatment with the NO donor nitroglycerine corrected the defective responses in NOS2 deficient mice. As presented in our recent study [105], in a mouse model of delayed fracture healing caused by periosteal cauterization, an absence of NOS2 or NOS3 resulted in a diminished bone formation with significantly lower bone volumes measured and a shift in these mice from delayed union towards nonunion. Comparable results were found in a fracture-healing study conducted by Kdolsky et al., were arginine was administered to guinea-pigs subjected to a 7 mm diaphyseal and periosteal femoral defect stabilized intramedullary with a K-wire. Radiographic analysis of these animals showed an increased number of healed fractures in the treatment group when compared to control animals [63].

Osteocalcin, a protein which is produced and secreted by osteoblasts, is increased in serum of rats fed L-NAME with/without addition of L-arginine via the drinking water for a period of 18 days. Serum levels reflect systemic bone formation; however, bone formation indices in tibial epiphysis in these rats showed no correlation with osteocalcin concentrations [93].

### 2.4. Possible Applications for D-Enantiomeric Amino Acids

In recent years, a possible role for D-amino acids had been investigated in bone research and fracture repair [106,107]. With the development of different fracture-healing animal models [108], mainly in mice and rats, the possibilities for research into infectious complications during bone healing have increased [109,110,111]. In the clinical situation there is still a high risk for developing implant-related infectious complications [112]. *Staphylococcus aureus* is the micro-organism which is most abundant in chronic osteomyelitis [113] and with a high potential of forming biofilms [114] and associated incidences of nonunion development [115]. Recent studies by Sanchez et al. showed that a local delivery of a combination of D-amino acids from biofilm-dispersive scaffolds showed a reduced *S. aureus* contamination in vivo and in vitro [106]. On the contrary, although in vitro experiments showed that D-amino acids also inhibit bone marrow stromal cell proliferation and differentiation of osteoblasts and osteoclasts, new bone formation in an ovine model is not hampered [107]. More research into the role of D-amino acids needs to be conducted to elucidate the so far contradicting results found in these studies.

## 3. Concluding Remarks and Future Possibilities

In this review, a large amount of preclinical evidence is presented to substantiate the hypothesis that in clinical development of nonunion, specific amino acid deficiencies play an important role. Mainly, the arginine-citrulline-nitric oxide metabolism has a major influence on the process of fracture repair, more specifically appropriate concentrations of amino acids and temporal expression of nitric oxide synthase enzymes are of utmost importance for an adequate bone-healing process. Future clinical research should focus on detecting specific amino acid deficiencies in patients directly after sustaining a fracture, and on the other hand on randomized controlled trials focusing on the results of amino acid supplementation in patients with observed deficiencies.

## References

[1] Kannus P., Niemi S., Palvanen M., Parkkari J., Pasanen M., Jarvinen M., Vuori I. (2001). Continuously rising problem of osteoporotic knee fractures in elderly women: Nationwide statistics in Finland in 1970–1999 and predictions until the year 2030. Bone.

[2] van Staa T.P., Dennison E.M., Leufkens H.G., Cooper C. (2001). Epidemiology of fractures in England and Wales. Bone.

[3] Johnell O., Kanis J. (2005). Epidemiology of osteoporotic fractures. Osteoporos. Int..

[4] Alves C.J., Neto E., Sousa D.M., Leitao L., Vasconcelos D.M., Ribeiro-Silva M., Alencastre I.S., Lamghari M. (2016). Fracture pain-Traveling unknown pathways. Bone.

[5] Knuth A.G., Hallal P.C. (2009). Temporal trends in physical activity: A systematic review. J. Phys. Act. Health.

[6] Mills L.A., Simpson A.H. (2013). The relative incidence of fracture non-union in the Scottish population (5.17 million): A 5-year epidemiological study. BMJ Open.

[7] Antonova E., Le T.K., Burge R., Mershon J. (2013). Tibia shaft fractures: Costly burden of nonunions. BMC Musculoskelet. Disord..

[8] Schindeler A., McDonald M.M., Bokko P., Little D.G. (2008). Bone remodeling during fracture repair: The cellular picture. Semin. Cell Dev. Biol..

[9] Gerstenfeld L.C., Cullinane D.M., Barnes G.L., Graves D.T., Einhorn T.A. (2003). Fracture healing as a post-natal developmental process: Molecular, spatial, and temporal aspects of its regulation. J. Cell. Biochem..

[10] Claes L., Recknagel S., Ignatius A. (2012). Fracture healing under healthy and inflammatory conditions. Nat. Rev. Rheumatol..

[11] Court-Brown C.M., McQueen M.M. (2008). Nonunions of the proximal humerus: Their prevalence and functional outcome. J. Trauma.

[12] Einhorn T.A. (1995). Enhancement of fracture-healing. J. Bone Jt. Surg. Am..

[13] Wijnands K.A., Brink P.R., Weijers P.H., Dejong C.H., Poeze M. (2012). Impaired fracture healing associated with amino acid disturbances. Am. J. Clin. Nutr..

[14] Bhandari M., Guyatt G.H., Swiontkowski M.F., Tornetta P., Sprague S., Schemitsch E.H. (2002). A lack of consensus in the assessment of fracture healing among orthopaedic surgeons. J. Orthop. Trauma.

[15] Frolke J.P., Patka P. (2007). Definition and classification of fracture non-unions. Injury.

[16] Megas P. (2005). Classification of non-union. Injury.

[17] Bishop J.A., Palanca A.A., Bellino M.J., Lowenberg D.W. (2012). Assessment of compromised fracture healing. J. Am. Acad. Orthop. Surg..

[18] Robinson C.M., Court-Brown C.M., McQueen M.M., Wakefield A.E. (2004). Estimating the risk of nonunion following nonoperative treatment of a clavicular fracture. J. Bone Jt. Surg. Am..

[19] Kozin S.H. (2001). Incidence, mechanism, and natural history of scaphoid fractures. Hand Clin..

[20] Rosenberg G.A., Sferra J.J. (2000). Treatment strategies for acute fractures and nonunions of the proximal fifth metatarsal. J. Am. Acad. Orthop. Surg..

[21] Allen M.R., Hock J.M., Burr D.B. (2004). Periosteum: Biology, regulation, and response to osteoporosis therapies. Bone.

[22] Roberts S.J., van Gastel N., Carmeliet G., Luyten F.P. (2015). Uncovering the periosteum for skeletal regeneration: The stem cell that lies beneath. Bone.

[23] Calori G.M., Albisetti W., Agus A., Iori S., Tagliabue L. (2007). Risk factors contributing to fracture non-unions. Injury.

[24] Gustilo R.B., Gruninger R.P., Davis T. (1987). Classification of type III (severe) open fractures relative to treatment and results. Orthopedics.

[25] Schmidmaier G., Capanna R., Wildemann B., Beque T., Lowenberg D. (2009). Bone morphogenetic proteins in critical-size bone defects: What are the options?. Injury.

[26] Hernandez R.K., Do T.P., Critchlow C.W., Dent R.E., Jick S.S. (2012). Patient-related risk factors for fracture-healing complications in the United Kingdom General Practice Research Database. Acta Orthop..

[27] Court-Brown C.M., Caesar B. (2006). Epidemiology of adult fractures: A review. Injury.

[28] Buhr A.J., Cooke A.M. (1959). Fracture patterns. Lancet.

[29] Brinker M.R., O’Connor D.P., Monla Y.T., Earthman T.P. (2007). Metabolic and endocrine abnormalities in patients with nonunions. J. Orthop. Trauma.

[30] MacKay D., Miller A.L. (2003). Nutritional support for wound healing. Altern. Med. Rev..

[31] Cunniffe G.M., Dickson G.R., Partap S., Stanton K.T., O’Brien F.J. (2010). Development and characterisation of a collagen nano-hydroxyapatite composite scaffold for bone tissue engineering. J. Mater. Sci. Mater. Med..

[32] Gross R.L. (2000). The effect of ascorbate on wound healing. Int. Ophthalmol. Clin..

[33] Oliveira S.M., Ringshia R.A., Legeros R.Z., Clark E., Yost M.J., Terracio L., Teixeira C.C. (2010). An improved collagen scaffold for skeletal regeneration. J. Biomed. Mater. Res. A.

[34] Nakase T., Yoshikawa H. (2006). Potential roles of bone morphogenetic proteins (BMPs) in skeletal repair and regeneration. J. Bone Miner. Metab..

[35] Urist M.R. (1965). Bone: Formation by autoinduction. Science.

[36] Axelrad T.W., Einhorn T.A. (2009). Bone morphogenetic proteins in orthopaedic surgery. Cytokine Growth Factor Rev..

[37] Cheng H., Jiang W., Phillips F.M., Haydon R.C., Peng Y., Zhou L., Luu H.H., An N., Breyer B., Vanichakarn P. (2003). Osteogenic activity of the fourteen types of human bone morphogenetic proteins (BMPs). J. Bone Jt. Surg. Am..

[38] Cho T.J., Gerstenfeld L.C., Einhorn T.A. (2002). Differential temporal expression of members of the transforming growth factor beta superfamily during murine fracture healing. J. Bone Miner. Res..

[39] Kloen P., Di Paola M., Borens O., Richmond J., Perino G., Helfet D.L., Goumans M.J. (2003). BMP signaling components are expressed in human fracture callus. Bone.

[40] Barnes G.L., Kostenuik P.J., Gerstenfeld L.C., Einhorn T.A. (1999). Growth factor regulation of fracture repair. J. Bone Miner. Res..

[41] Carreira A.C., Lojudice F.H., Halcsik E., Navarro R.D., Sogayar M.C., Granjeiro J.M. (2014). Bone morphogenetic proteins: Facts, challenges, and future perspectives. J. Dent. Res..

[42] Bustos-Valenzuela J.C., Halcsik E., Bassi E.J., Demasi M.A., Granjeiro J.M., Sogayar M.C. (2010). Expression, purification, bioactivity, and partial characterization of a recombinant human bone morphogenetic protein-7 produced in human 293T cells. Mol. Biotechnol..

[43] Giannoudis P.V., Gudipati S., Harwood P., Kanakaris N.K. (2015). Long bone non-unions treated with the diamond concept: A case series of 64 patients. Injury.

[44] Calori G.M., Mazza E., Colombo M., Ripamonti C., Tagliabue L. (2011). Treatment of long bone non-unions with polytherapy: Indications and clinical results. Injury.

[45] Secreto F.J., Hoeppner L.H., Westendorf J.J. (2009). Wnt signaling during fracture repair. Curr. Osteoporos. Rep..

[46] Westendorf J.J., Kahler R.A., Schroeder T.M. (2004). Wnt signaling in osteoblasts and bone diseases. Gene.

[47] Ali A., Hoeflich K.P., Woodgett J.R. (2001). Glycogen synthase kinase-3: Properties, functions, and regulation. Chem. Rev..

[48] Bao Q., Chen S., Qin H., Feng J., Liu H., Liu D., Li A., Shen Y., Zhao Y., Li J. (2017). An appropriate Wnt/beta-catenin expression level during the remodeling phase is required for improved bone fracture healing in mice. Sci. Rep..

[49] Deutz N.E., Matheson E.M., Matarese L.E., Luo M., Baggs G.E., Nelson J.L., Hegazi R.A., Tappenden K.A., Ziegler T.R. (2016). Readmission and mortality in malnourished, older, hospitalized adults treated with a specialized oral nutritional supplement: A randomized clinical trial. Clin. Nutr..

[50] Koski K., Luukinen H., Laippala P., Kivela S.L. (1998). Risk factors for major injurious falls among the home-dwelling elderly by functional abilities. A prospective population-based study. Gerontology.

[51] Cederholm T., Hedstrom M. (2005). Nutritional treatment of bone fracture. Curr. Opin. Clin. Nutr. Metab. Care.

[52] Lill C.A., Hesseln J., Schlegel U., Eckhardt C., Goldhahn J., Schneider E. (2003). Biomechanical evaluation of healing in a non-critical defect in a large animal model of osteoporosis. J. Orthop. Res..

[53] Neyens J., Halfens R., Spreeuwenberg M., Meijers J., Luiking Y., Verlaan G., Schols J. (2013). Malnutrition is associated with an increased risk of falls and impaired activity in elderly patients in Dutch residential long-term care (LTC): A cross-sectional study. Arch. Gerontol. Geriatr..

[54] Lim S.L., Ong K.C., Chan Y.H., Loke W.C., Ferguson M., Daniels L. (2012). Malnutrition and its impact on cost of hospitalization, length of stay, readmission and 3-year mortality. Clin. Nutr..

[55] Oakland K., Nadler R., Cresswell L., Jackson D., Coughlin P.A. (2016). Systematic review and meta-analysis of the association between frailty and outcome in surgical patients. Ann. R. Coll. Surg. Engl..

[56] Sullivan D.H., Sun S., Walls R.C. (1999). Protein-energy undernutrition among elderly hospitalized patients: A prospective study. JAMA.

[57] Allard J.P., Keller H., Jeejeebhoy K.N., Laporte M., Duerksen D.R., Gramlich L., Payette H., Bernier P., Vesnaver E., Davidson B. (2015). Malnutrition at Hospital Admission-Contributors and Effect on Length of Stay: A Prospective Cohort Study From the Canadian Malnutrition Task Force. JPEN J. Parenter. Enter. Nutr..

[58] Wall B.T., Dirks M.L., Snijders T., Senden J.M., Dolmans J., van Loon L.J. (2014). Substantial skeletal muscle loss occurs during only 5 days of disuse. Acta Physiol..

[59] Cawthon P.M., Blackwell T.L., Cauley J., Kado D.M., Barrett-Connor E., Lee C.G., Hoffman A.R., Nevitt M., Stefanick M.L., Lane N.E. (2015). Evaluation of the Usefulness of Consensus Definitions of Sarcopenia in Older Men: Results from the Observational Osteoporotic Fractures in Men Cohort Study. J. Am. Geriatr. Soc..

[60] Zura R., Braid-Forbes M.J., Jeray K., Mehta S., Einhorn T.A., Watson J.T., Della Rocca G.J., Forbes K., Steen R.G. (2017). Bone fracture nonunion rate decreases with increasing age: A prospective inception cohort study. Bone.

[61] Poeze M., Bruins M.J., Kessels F., Luiking Y.C., Lamers W.H., Deutz N.E. (2011). Effects of L-arginine pretreatment on nitric oxide metabolism and hepatosplanchnic perfusion during porcine endotoxemia. Am. J. Clin. Nutr..

[62] Wijnands K.A., Vink H., Briede J.J., van Faassen E.E., Lamers W.H., Buurman W.A., Poeze M. (2012). Citrulline a More Suitable Substrate than Arginine to Restore NO Production and the Microcirculation during Endotoxemia. PLoS ONE.

[63] Kdolsky R.K., Mohr W., Savidis-Dacho H., Beer R., Puig S., Reihsner R., Tangl S., Donath K. (2005). The influence of oral L-arginine on fracture healing: An animal study. Wien. Klin. Wochenschr..

[64] Angele M.K., Fitzal F., Smail N., Knoferl M.W., Schwacha M.G., Ayala A., Wang P., Chaudry I.H. (2000). L-arginine attenuates trauma-hemorrhage-induced liver injury. Crit. Care Med..

[65] Luiking Y.C., Poeze M., Deutz N.E. (2015). Arginine infusion in patients with septic shock increases nitric oxide production without haemodynamic instability. Clin. Sci..

[66] van Wijck K., Wijnands K.A., Meesters D.M., Boonen B., van Loon L.J., Buurman W.A., Dejong C.H., Lenaerts K., Poeze M. (2014). L-citrulline improves splanchnic perfusion and reduces gut injury during exercise. Med. Sci. Sports Exerc..

[67] Jones M.S., Rivera M., Puccinelli C.L., Wang M.Y., Williams S.J., Barber A.E. (2014). Targeted amino acid supplementation in diabetic foot wounds: Pilot data and a review of the literature. Surg. Infect..

[68] Luiking Y.C., Poeze M., Ramsay G., Deutz N.E. (2009). Reduced citrulline production in sepsis is related to diminished de novo arginine and nitric oxide production. Am. J. Clin. Nutr..

[69] Daly J.M., Reynolds J., Thom A., Kinsley L., Dietrick-Gallagher M., Shou J., Ruggieri B. (1988). Immune and metabolic effects of arginine in the surgical patient. Ann. Surg..

[70] Evans C.H., Stefanovic-Racic M., Lancaster J. (1995). Nitric oxide and its role in orthopaedic disease. Clin. Orthop. Relat. Res..

[71] Long C.L., Geiger J.W., Richards E.W., Akin J.M., Blakemore W.S. (1992). Plasma amino acid concentrations in geriatric control and hip-fracture patients. Am. J. Clin. Nutr..

[72] Woolf L.I., Grovers A.C., Moore J.P., Duff J.H., Finley R.J., Loomer R.L. (1976). Arterial plasma amino acids in patients with serious postoperative infection and in patients with major fractures. Surgery.

[73] Vittur F., Lunazzi G., Moro L., Stagni N., de Bernard B., Moretti M., Stanta G., Bacciottini F., Orlandini G., Reali N. (1986). A possible role for polyamines in cartilage in the mechanism of calcification. Biochim. Biophys. Acta.

[74] Patterson B.M., Cornell C.N., Carbone B., Levine B., Chapman D. (1992). Protein depletion and metabolic stress in elderly patients who have a fracture of the hip. J. Bone Jt. Surg. Am..

[75] Fini M., Aldini N.N., Cane V., Zaffe D., Giavaresi G., Rocca M., Guzzardella G.A., Giardino R. (1999). Effects of essential amino acids and lactose on bony fractures and defects in rabbits: A preliminary histomorphometric study. Arch. Orthop. Trauma Surg..

[76] Volpi E., Kobayashi H., Sheffield-Moore M., Mittendorfer B., Wolfe R.R. (2003). Essential amino acids are primarily responsible for the amino acid stimulation of muscle protein anabolism in healthy elderly adults. Am. J. Clin. Nutr..

[77] De Bandt J.P., Cynober L.A. (1998). Amino acids with anabolic properties. Curr. Opin. Clin. Nutr. Metab. Care.

[78] Hughes M.S., Kazmier P., Burd T.A., Anglen J., Stoker A.M., Kuroki K., Carson W.L., Cook J.L. (2006). Enhanced fracture and soft-tissue healing by means of anabolic dietary supplementation. J. Bone Jt. Surg. Am..

[79] Day S.M., DeHeer D.H. (2001). Reversal of the detrimental effects of chronic protein malnutrition on long bone fracture healing. J. Orthop. Trauma.

[80] Torricelli P., Fini M., Giavaresi G., Giardino R. (2003). Human osteopenic bone-derived osteoblasts: Essential amino acids treatment effects. Artif. Cells Blood Substit. Immobil. Biotechnol..

[81] Chevalley T., Rizzoli R., Manen D., Caverzasio J., Bonjour J.P. (1998). Arginine increases insulin-like growth factor-I production and collagen synthesis in osteoblast-like cells. Bone.

[82] Trippel S.B. (1998). Potential role of insulinlike growth factors in fracture healing. Clin. Orthop. Relat. Res..

[83] Fini M., Torricelli P., Giavaresi G., Carpi A., Nicolini A., Giardino R. (2001). Effect of L-lysine and L-arginine on primary osteoblast cultures from normal and osteopenic rats. Biomed. Pharmacother..

[84] Diwan A.D., Wang M.X., Jang D., Zhu W., Murrell G.A. (2000). Nitric oxide modulates fracture healing. J. Bone Miner. Res..

[85] Schaffer M.R., Tantry U., Gross S.S., Wasserburg H.L., Barbul A. (1996). Nitric oxide regulates wound healing. J. Surg. Res..

[86] Murrell G.A., Szabo C., Hannafin J.A., Jang D., Dolan M.M., Deng X.H., Murrell D.F., Warren R.F. (1997). Modulation of tendon healing by nitric oxide. Inflamm. Res..

[87] Sakurai H., Kohsaka H., Liu M.F., Higashiyama H., Hirata Y., Kanno K., Saito I., Miyasaka N. (1995). Nitric oxide production and inducible nitric oxide synthase expression in inflammatory arthritides. J. Clin. Investig..

[88] Stefanovic-Racic M., Meyers K., Meschter C., Coffey J.W., Hoffman R.A., Evans C.H. (1994). *N*-monomethyl arginine, an inhibitor of nitric oxide synthase, suppresses the development of adjuvant arthritis in rats. Arthritis Rheum..

[89] Wimalawansa S.J., De Marco G., Gangula P., Yallampalli C. (1996). Nitric oxide donor alleviates ovariectomy-induced bone loss. Bone.

[90] Hukkanen M., Corbett S.A., Batten J., Konttinen Y.T., McCarthy I.D., Maclouf J., Santavirta S., Hughes S.P., Polak J.M. (1997). Aseptic loosening of total hip replacement. Macrophage expression of inducible nitric oxide synthase and cyclo-oxygenase-2, together with peroxynitrite formation, as a possible mechanism for early prosthesis failure. J. Bone Jt. Surg. Br..

[91] Xia W., Szomor Z., Wang Y., Murrell G.A. (2006). Nitric oxide enhances collagen synthesis in cultured human tendon cells. J. Orthop. Res..

[92] Ekeland A., Engesaeter L.B., Langeland N. (1981). Mechanical properties of fractured and intact rat femora evaluated by bending, torsional and tensile tests. Acta Orthop. Scand..

[93] Turner C.H., Owan I., Jacob D.S., McClintock R., Peacock M. (1997). Effects of nitric oxide synthase inhibitors on bone formation in rats. Bone.

[94] Shabani M., Pulfer S.K., Bulgrin J.P., Smith D.J. (1996). Enhancement of wound repair with a topically applied nitric oxide-releasing polymer. Wound Repair Regen..

[95] Yamasaki K., Edington H.D., McClosky C., Tzeng E., Lizonova A., Kovesdi I., Steed D.L., Billiar T.R. (1998). Reversal of impaired wound repair in iNOS-deficient mice by topical adenoviral-mediated iNOS gene transfer. J. Clin. Investig..

[96] Baldik Y., Diwan A.D., Appleyard R.C., Fang Z.M., Wang Y., Murrell G.A. (2005). Deletion of iNOS gene impairs mouse fracture healing. Bone.

[97] Zhu W., Diwan A.D., Lin J.H., Murrell G.A. (2001). Nitric oxide synthase isoforms during fracture healing. J. Bone Miner. Res..

[98] Einhorn T.A., Majeska R.J., Rush E.B., Levine P.M., Horowitz M.C. (1995). The expression of cytokine activity by fracture callus. J. Bone Miner. Res..

[99] Hikiji H., Shin W.S., Oida S., Takato T., Koizumi T., Toyo-oka T. (1997). Direct action of nitric oxide on osteoblastic differentiation. FEBS Lett..

[100] Zhu W., Murrell G.A., Lin J., Gardiner E.M., Diwan A.D. (2002). Localization of nitric oxide synthases during fracture healing. J. Bone Miner. Res..

[101] Pilla A.A. (2013). Nonthermal electromagnetic fields: From first messenger to therapeutic applications. Electromagn. Biol. Med..

[102] Turner C.H., Takano Y., Owan I., Murrell G.A. (1996). Nitric oxide inhibitor L-NAME suppresses mechanically induced bone formation in rats. Am. J. Physiol..

[103] de Albuquerque R.F., Del Bel E.A., Brentegani L.G., Moura de Oliveira M.T., Issa J.P.M. (2008). Trigeminal nitric oxide synthase expression correlates with new bone formation during distraction osteogenesis. Calcif. Tissue Int..

[104] Watanuki M., Sakai A., Sakata T., Tsurukami H., Miwa M., Uchida Y., Watanabe K., Ikeda K., Nakamura T. (2002). Role of inducible nitric oxide synthase in skeletal adaptation to acute increases in mechanical loading. J. Bone Miner. Res..

[105] Meesters D.M., Neubert S., Wijnands K.A., Heyer F.L., Zeiter S., Ito K., Brink P.R., Poeze M. (2016). Deficiency of inducible and endothelial nitric oxide synthase results in diminished bone formation and delayed union and nonunion development. Bone.

[106] Sanchez C.J., Prieto E.M., Krueger C.A., Zienkiewicz K.J., Romano D.R., Ward C.L., Akers K.S., Guelcher S.A., Wenke J.C. (2013). Effects of local delivery of d-amino acids from biofilm-dispersive scaffolds on infection in contaminated rat segmental defects. Biomaterials.

[107] Harmata A.J., Ma Y., Sanchez C.J., Zienkiewicz K.J., Elefteriou F., Wenke J.C., Guelcher S.A. (2015). d-amino acid inhibits biofilm but not new bone formation in an ovine model. Clin. Orthop. Relat. Res..

[108] Holstein J.H., Garcia P., Histing T., Kristen A., Scheuer C., Menger M.D., Pohlemann T. (2009). Advances in the establishment of defined mouse models for the study of fracture healing and bone regeneration. J. Orthop. Trauma.

[109] Inzana J.A., Schwarz E.M., Kates S.L., Awad H.A. (2015). A novel murine model of established Staphylococcal bone infection in the presence of a fracture fixation plate to study therapies utilizing antibiotic-laden spacers after revision surgery. Bone.

[110] Reizner W., Hunter J.G., O’Malley N.T., Southgate R.D., Schwarz E.M., Kates S.L. (2014). A systematic review of animal models for Staphylococcus aureus osteomyelitis. Eur. Cells Mater..

[111] Li D., Gromov K., Soballe K., Puzas J.E., O’Keefe R.J., Awad H., Drissi H., Schwarz E.M. (2008). Quantitative mouse model of implant-associated osteomyelitis and the kinetics of microbial growth, osteolysis, and humoral immunity. J. Orthop. Res..

[112] Darouiche R.O. (2004). Treatment of infections associated with surgical implants. N. Engl. J. Med..

[113] Brady R.A., Leid J.G., Calhoun J.H., Costerton J.W., Shirtliff M.E. (2008). Osteomyelitis and the role of biofilms in chronic infection. FEMS Immunol. Med. Microbiol..

[114] Costerton J.W., Montanaro L., Arciola C.R. (2005). Biofilm in implant infections: Its production and regulation. Int. J. Artif. Organs.

[115] Palmer M., Costerton W., Sewecke J., Altman D. (2011). Molecular techniques to detect biofilm bacteria in long bone nonunion: A case report. Clin. Orthop. Relat. Res..

